# Usability of the Turkish Translation of the Dutch Talking Touch Screen Questionnaire for Physical Therapy Patients With a Turkish Background: Qualitative Study

**DOI:** 10.2196/14189

**Published:** 2020-02-13

**Authors:** Marlies Welbie, Harriet Wittink, Sahin Bozkurt, Tugba Coban, Walter LJM Devillé

**Affiliations:** 1 Research Group Lifestyle and Health Research Center Healthy and Sustainable Living Utrecht University of Applied Sciences Utrecht Netherlands; 2 Julius Centre for health Sciences and Primary Care University Medical Centre Utrecht Utrecht Netherlands; 3 Faculty of Social and Behavioral Sciences University of Amsterdam Amsterdam Netherlands; 4 Dutch Centre of Expertise on Health Disparities (Pharos) Utrecht Netherlands

**Keywords:** mHealth, eHealth, surveys and questionnaires, physical therapy specialty, qualitative research

## Abstract

**Background:**

The Turkish translation of the Dutch Talking Touch Screen Questionnaire (TTSQ) has been developed to help physical therapy patients with a Turkish background in the Netherlands to autonomously elucidate their health problems and impairments and set treatment goals, regardless of their level of health literacy.

**Objective:**

The aim of this study was to evaluate the usability of the Turkish TTSQ for physical therapy patients with a Turkish background with diverse levels of health literacy and experience in using mobile technology.

**Methods:**

The qualitative Three-Step Test-Interview method was carried out to gain insight into the usability of the Turkish TTSQ. A total of 10 physical therapy patients participated. The interview data were analyzed using a thematic content analysis approach aimed at determining the accuracy and completeness with which participants completed the questionnaire (effectiveness), the time it took participants to complete the questionnaire (efficiency), and the extent to which the participants were satisfied with the ease of use of the questionnaire (satisfaction). The problems encountered by the participants in this study were given a severity rating, which was used to provide a rough estimate of the need for additional usability improvements.

**Results:**

No participant in this study was able to complete the questionnaire without encountering at least one usability problem. A total of 17 different kinds of problems were found. On the basis of their severity score, 3 problems that should be addressed during future development of the tool were “Not using the navigation function of the photo gallery in Question 4 causing the participant to not see all presented response items;” “Touching the text underneath a photo in Question 4 to select an activity instead of touching the photo itself, causing the activity not to be selected;” and “Pushing too hard or tapping too softly on the touch screen causing the touch screen to not respond.” The data on efficiency within this study were not valid and are, therefore, not reported in this study. No participant was completely satisfied or dissatisfied with the overall ease of use of the Turkish TTSQ. Two participants with no prior experience of using tablet computers felt that, regardless of what kinds of improvement might be made, it would just be too difficult for them to learn to work with the device.

**Conclusions:**

As with the Dutch TTSQ, the Turkish TTSQ needs improvement before it can be released. The results of this study confirm the conclusion of the Dutch TTSQ study that participants with low levels of education and little experience in using mobile technology are less able to operate the TTSQ effectively. Using a Dutch speaking interviewer and Turkish interpreter has had a negative effect on data collection in this study.

## Introduction

### Background

In the past three decades, health care provision in the Netherlands has evolved from a paternalistic to a patient-centered care approach. Since 1995, the government has introduced a series of laws and regulations aimed at increasing the autonomy and self-determination of patients [1]. Even today, policy makers, institutions, and health care professionals strive to further develop shared decision making and self-management in patients. Patients are increasingly expected to behave as active partners in encounters with health care professionals [2]. Not all patients are able to take on such a role. An important undermining factor is inadequate health literacy [3-5], which applies to 36% of the Dutch population [6].

Health literacy is defined as the cognitive and social skills that determine the motivation and ability of individuals to gain access to, understand, and use information in ways which promote and maintain health [7]. The concept contains cognitive and noncognitive aspects [8]. Cognitive aspects are referred to as “the capacity to think” and comprise functional skills such as literacy, numeracy, and information processing. Noncognitive aspects are referred to as “the capacity to act” and comprise skills such as goal setting, making a plan, and taking action [9]. Having the capacity to think and to act are equally important preconditions for patients taking on a proactive role during encounters with health professionals [8]. The majority of health literacy interventions, however, are aimed at improving cognitive skills [10-18]. To create a successful health literacy intervention, developers should (1) try to best fit the needs of persons with inadequate health literacy by incorporating members of the target group into their design team and (2) focus on noncognitive, as well as cognitive, aspects of health literacy [11]. On the basis of the results of current research, the possibilities of training noncognitive skills are expected to be limited [9]. This may mean that interventions aimed at increasing “the capacity to act” should not be focused on *training* noncognitive skills but on *supporting* them. This was exactly what the initiators of the development of the Dutch Talking Touch Screen Questionnaire (TTSQ) had in mind [19].

The Dutch TTSQ has been developed to help Dutch physical therapy patients, regardless of their level of health literacy, to elucidate their health problems and impairments, and set treatment goals. A total of 10 low-literate persons were involved in the development process of the prototype. In this prototype, which runs on a tablet computer, plain language and self-explanatory scales were used, alternatives to text were offered (eg, audio, pictures, and clips), and easily accessible background information on the questionnaire’s rationale was provided. The development of the prototype of the Dutch TTSQ was described in detail in the study by Cremers et al [19]. It was pretested for usability [20] and face validity [21]. The results of both studies were promising but showed the need for further development.

Alongside the Dutch version, a Turkish version was developed. Development of this was seen as a starting point for development of other language versions. The initiators started with the Turkish version because people with a Turkish background form the biggest minority group in the Netherlands (about 400,000 people, 2.3% of the total population) [22]. Approximately one-third of the Turkish people aged between 15 and 65 years in the Netherlands only went to primary school, compared with 6% of the Dutch majority population [23]. The proportion of Turkish people with low literacy and low health literacy is unknown but, as education and literacy are very strongly associated [24,25], one can assume that low literacy and low health literacy are overrepresented in the Turkish minority group. Most people with low literacy are not digitally skilled [26], and recent studies found ethnic and socioeconomic differences in the use of mobile technology [27,28]. Therefore, it is to be expected that a relatively large proportion of this target population has little experience of using mobile technology. This may be a complicating factor in the use of the Turkish version of the TTSQ.

### Objective

The aim of this study was to test the prototype of the Turkish TTSQ within the physical therapy context to see which parts of the prototype needed adjustment to increase user-friendliness for physical therapy patients with a Turkish background, regardless of their level of health literacy or experience of operating mobile technology.

The research question underlying this study was “What is the usability of the prototype of the Turkish TTSQ for physical therapy patients with a Turkish background with diverse levels of health literacy and experience in using mobile technology?”

## Methods

### Design

A qualitative descriptive case study [29] was carried out. Data were collected and analyzed as in the study on ease of use of the Dutch version of the TTSQ [20]. Data on the way participants operated the Turkish TTSQ were collected through the Three-Step Test-Interview (TSTI) method [30]. This method includes both think-aloud and retrospective probing techniques.

### Definitions

*Usability* was defined by the International Standards Organization as “the effectiveness, efficiency and satisfaction with which specified users can achieve goals in particular environments” [31].

*Effectiveness* is the accuracy and completeness with which users achieve certain goals [32]. In this study, rates and severity of problems were used as primary indicators of effectiveness.

*Efficiency* is the relation between the accuracy and completeness with which users achieve certain goals and the resources expended in achieving them [32]. In this study, task completion time was used as an indicator of efficiency.

*Satisfaction* is the users’ comfort with and positive attitudes toward the use of a system [32]. In this study, participants were interviewed about their satisfaction with the ease of use of the Turkish TTSQ. Ease of use was defined as the degree to which the use of a particular system is free from effort [33].

### Setting and Participant Selection

Recruitment took place in 12 primary care practices in deprived areas of Utrecht, the Netherlands. Potential participants were invited by their physical therapist to participate in this study. Researcher SB was a native Turkish speaker with a Turkish background and employed as a physical therapist in one of the recruiting practices. No other recruiting therapists had Turkish backgrounds or spoke Turkish. Each recruiting therapist shortly explained the goal of the study to potential participants and provided them with Turkish and Dutch versions of a flyer and information letter. The flyer contained a brief summary of the background and goal of the research project and an invitation to its readers to read more about the project in the accompanying information letter. Both versions of the flyer and information letter were written in plain language. If patients were interested in participating, their therapist asked permission to give their contact information to the researchers. If patients spoke and understood Dutch, researcher MW contacted them by telephone; otherwise, researcher SB contacted them. During the telephone conversation, the researchers invited questions, checked that patients understood what was being asked of them, and checked that inclusion criteria were met. Inclusion criteria were: aged 18 years or older, able to understand the Turkish language, and both parents born in Turkey. The sampling procedure was aimed at getting a sample of 6 to 12 participants, typical for formative usability testing of devices such as the TTSQ [34] because it would reveal the most important points needing improvement for further development of a tool without the risk of unnecessary expenditures [35]. Data collection was stopped when a good balance was reached in terms of age, gender, level of education, level of functional health literacy, and prior experience with using a tablet computer. Throughout the recruitment process, the recruiting physical therapists were constantly kept informed about the profiles of participants the researchers were looking for.

### Content of the Turkish Talking Touch Screen Questionnaire

The prototype of the Turkish TTSQ (see Multimedia Appendix 1) is a direct translation of the Dutch TTSQ [19-21], which is described in detail in the methodological sections by Welbie et al 2018 and 2019 [20,21].

Translation of the Dutch TTSQ into Turkish was done by a native Turkish speaker who worked as a Turkish language teacher in the Netherlands. Comprehension of the translated text was tested by researcher TC, a native Turkish speaker with a Turkish background. She asked 7 non-Dutch–speaking women, who were born in Turkey and now lived in the Netherlands, to read the written text, listen to the spoken text in the Turkish TTSQ, and explain to her what they thought was meant by the questions and answer options. The 7 women had finished primary school at most and were following different kinds of courses (such as cooking and handicraft) together at a mosque in Utrecht. The 7 Turkish female testers had no problems understanding both spoken and written text. An overview of all types of screens is given in Screenshots 1 to 16 in the Multimedia Appendix 1. The 8 questions of the questionnaire can be found in Multimedia Appendix 1: screenshots 2, 3, 4, 7, 9, 11, 12, and 13.

### Data Collection and Procedures

Data collection took place at the physical therapy practice or the participant’s home, depending on the preference of the participant. Researchers MW and SB were present. Researcher MW was in the lead during the interviews. She communicated in Dutch during the whole meeting. Researcher SB functioned as an observer as well as an interpreter when participants spoke Turkish. As an interpreter-researcher, SB did not interfere in the conversation but solely acted as an intermediary. Participants spoke Dutch, Turkish, or a mixture of both languages, depending on their preference and abilities. At the end of the interview, researcher SB asked complementary questions if some information was lacking. When SB asked these questions in Turkish, he directly translated them and later the answers given by the participants into Dutch so that researcher MW could closely follow what was said.

The following data-gathering steps were taken according to the TSTI method [30]:

- *Step 1:* All participants were observed by researchers MW and SB while they were completing the Turkish TTSQ. During the completion of the questionnaire, they thought out loud. When participants spoke Turkish or used some Turkish words, researcher SB took on the role of interpreter and translated the text into Dutch. This step was aimed at collecting observational data on the usability of the Turkish TTSQ. The data collected consisted of 2 types: (1) observations of participants’ behavior and (2) think-aloud data. A video recording was made of this interview step. The video camera was aimed at the tablet computer and the hands of the participant while operating the screen. In addition, both researchers MW and SB took real-time notes for use during the following steps of the interview as well as for later analysis. The researchers wrote their notes down on hard copies of screenshots of the Dutch TTSQ, which were printed next to the identical screens of the Dutch questionnaire, so researcher MW was able to read the question and answer options in Dutch. Researchers MW and SB noted problems with operating the tablet computer, including using the touch screen, navigating through the questionnaire, understanding the task given in each screen, selecting response items, using the correction function, and use of the stop and help buttons.
- *Step 2:* Researcher MW interviewed each participant after they had finished completing the Turkish TTSQ. Data collection during this step was exclusively focused on filling possible gaps and checking the observational data collected during step 1. An audio recording was made of this interview step.
- *Step 3:* During step 3 of the TSTI, researcher MW conducted a semistructured interview aimed at eliciting experiences and opinions of participants. At the end of the interview, researcher SB asked complementary questions, if he felt it was necessary, to get complete and rich data. When participants encountered problems in operating the Turkish TTSQ, they were asked what they thought the exact nature and possible cause of each type of problem was. In addition, they were asked how they tried to overcome the problem and if they had suggestions for making it easier to operate the Turkish TTSQ at this point. Afterwards, the participants were questioned about their satisfaction regarding the overall ease of use of the Turkish TTSQ. The participants were encouraged to report feelings, express opinions, state preferences, and make recommendations. An audio recording was made of this interview step.

When the interview was finished, demographic data, data on self-reported experience with using a tablet computer, self-reported health, and functional health literacy measured with the Set of Brief Screening Questions-Dutch version (SBSQ-D) [36] were collected (see Tables 1 and 2). The SBSQ-D is the Dutch version of Chew’s SBSQ. This tool consists of the following 3 statements: “How often do you have someone help you read hospital materials?” “How confident are you filling out medical forms by yourself?” and “How often do you have problems learning about your medical condition because of difficulty understanding written information?” The combined item-responses result in a subjective health literacy score [37,38]. The SBSQ-D was conducted orally by researcher SB who translated the statements into Turkish if necessary.

### Analyses

Data were analyzed using a thematic content analysis approach [39]. A total of 4 types of data were analyzed, which were as follows: (1) video recordings of the completion of the questionnaire, (2) field notes of the observed participant behavior, (3) transcriptions of the Dutch spoken text within the video and audio recordings, and (4) background information regarding educational level, level of literacy, age, gender, and prior experience using a tablet computer.

Only the *Dutch* spoken text within the interviews was transcribed. After transcription, researcher TC listened closely to the recordings while looking at the transcriptions of the Dutch spoken text. When she disagreed with the translation made by researcher SB during the interview, she added what she thought was a more accurate translation to the transcript in a different color. Afterwards, researcher TC and SB sought consensus on the most accurate translation.

Researcher MW started the coding process by coding step 1 of the interview directly on the video recordings, using MAXQDA 12 (VERBI Software). This was partly an inductive and partly a deductive process. The deductive process consisted of using the descriptions of the 13 usability problems found in the ease of use study of the Dutch TTSQ [20] as codes. The inductive process comprised open coding of new problems, statements of the participants about the cause of these problems, and the way they thought these problems could be avoided in the future. In addition, statements of participants about satisfaction regarding the ease of use of the Turkish TTSQ were coded, and completion times were registered. After researcher MW finished coding step 1 for 1 interview, she checked from the transcription of steps 2 and 3 of that interview whether the problems were described and spoken about in a way congruent with her analysis of step 1. If not congruent, she watched the video again to see if her initial coding for step 1 needed adjustment. In addition, she coded the statements participants made during steps 2 and 3 about the causes of problems during completion of the Turkish TTSQ and the ways they thought these problems could be avoided. She also coded all statements of participants about satisfaction with ease of use of the Turkish TTSQ.

Directly after coding all 3 parts of an interview, researcher MW made a descriptive summary of that interview. Each summary contained information on whether or not the questionnaire was fully completed; if, when and why the stop function was used; if, when and why the help function was used and whether this was effective; the kinds of problems that occurred with the operation; the completion times; and all emerging themes regarding satisfaction with ease of use of the questionnaire. The themes emerging in the summaries were supplemented with related field notes and information regarding educational level, health literacy level, age, gender, and experience in using mobile technology. Afterwards, researcher MW compared this summary with that made at the end of the interview to check for inconsistencies. If any were found, she looked at all related data again to see if her interpretation and coding of what had happened and was said during the interview needed adjustment.

As the last step of the content analysis, researcher SB took on the role of peer debriefer to test the emerged hypotheses and see if they were reasonable and plausible to him. To get a good understanding of how the hypotheses emerged, researchers MW and SB looked at the summaries, codes, and raw data (transcripts and videos) together. During their conversation, they constantly and explicitly reflected on the influence their Turkish and Dutch backgrounds might have had on their views on the data and whether or not this made their interpretations of the data differ at any point.

As a next step, researcher MW extracted the observed usability problems from the summaries. MW reanalyzed the video recordings to see how many times each problem had occurred in total and per participant. After a full overview of problems had emerged, she categorized the problems as low, medium, serious, or critical as described by Nielsen and Loranger [40]. The scoring method was described in detail in Welbie et al [20]. Nielsen and Loranger recommend tackling only serious and critical problems during the development of a digital tool because those of low and medium severity are not worth tackling from a cost-benefit perspective. Serious and critical problems, however, can be so disruptive that they make users stop using a tool or prevent them from even starting to use it [40].

During the whole course of the study, procedures, coding, analysis steps, and interpretation decisions were discussed with researchers HW and WD.

Transcripts were made in the Dutch language. Only quotes used in this paper were translated from Dutch into English by researcher MW and checked by researcher HW, who is a bilingual speaker.

### Ethics

No external funding was received by the Utrecht University of Applied Sciences to conduct this study. This study was registered with the medical ethics committee of the Academic Medical Centre of Amsterdam, which declared that it does not fall under the scope of the “Medical Research Involving Human Subjects Act.” The study was conducted according to the principles of the Declaration of Helsinki [41]. All participants provided written informed consent. The participants’ names used in this study are all fictitious to protect their privacy.

## Results

### Study Population

A total of 10 physical therapy patients were included in this study. Characteristics of the study population can be found in Tables 1 and 2.

**Table 1:** Characteristics of study population (n=10).

Characteristics		Value
Age (years), mean (range)		53 (35-74)
**Gender, n**		
	Male	6
	Female	4
**Level of education, n**		
	Low^a^	4
	Moderate^b^	4
	High^c^	2
**Functional health literacy level measured with** **Set of Brief Screening Questions-Dutch version** **[36]**		
	Adequate	5
	Inadequate	5
**Prior experience operating a tablet computer, n**		
	Yes	5
	No	5

^a^Low: none or at most finished primary education.

^b^Moderate: lower secondary education, (upper) secondary education, or post-secondary nontertiary education (including vocational education).

^c^High: tertiary education (bachelor’s degree or higher).

**Table 2:** Characteristics per participant.

Pseudonym	Gender (F: female and M: male)	Age (years)	Level of education	Functional health literacy level measured with Set of Brief Screening Questions-Dutch version [36]	Self-reported health status	Prior experience using a tablet computer
Meryem	F	74	Low^a^	Inadequate	Poor	No
Mert	M	71	Low^a^	Inadequate	Poor	No
Ceyda	F	65	Low^a^	Inadequate	Satisfactory	No
Gizem	F	44	Low^a^	Inadequate	Poor	Yes
Memhet	M	59	Moderate^b^	Inadequate	Good	No
Berat	M	38	Moderate^b^	Adequate	Satisfactory	Yes
Elif	F	40	Moderate^b^	Adequate	Good	Yes
Eren	M	48	Moderate^b^	Adequate	Good	No
Imraam	M	52	High^c^	Adequate	Good	Yes
Onur	M	35	High^c^	Adequate	Good	Yes

^a^Low: none or at most finished primary education.

^b^Moderate: lower secondary education, (upper) secondary education, or postsecondary nontertiary education (including vocational education).

^c^High: tertiary education (bachelor’s degree or higher).

### Effectiveness

Of the 10 participants, 2 managed to complete the questionnaire fully. Both had prior experience with operating tablet computers (see Table 3). Ceyda (age 65 years) and Meryem (age 74 years) left all questions open, and Mehmet (age 59 years) stopped completing the questionnaire at question 5. All 3 were inexperienced in operating tablet computers. Inexperienced Eren (age 48 years) and Mert (age 71 years) and experienced Imraam (age 52 years), Elif (age 40 years), and Gizem (age 44 years) went through the whole questionnaire but unintentionally left 1 or more parts incomplete.

**Table 3:** Prior experience with using a tablet computer in comparison with ability to fully complete the Turkish Talking Touch Screen Questionnaire.

Population	Not fully completed, n	Fully completed, n
No prior experience using a tablet computer (n=5)	5	0
Prior experience using a tablet computer (n=5)	3	2
Total population (n=10)	8	2

### Unintentionally Unanswered (Parts of) Questions

Inexperienced Eren (age 48 years) and Mert (age 71 years) and experienced Imraam (age 52 years), Elif (age 40 years), and Gizem (age 44 years) failed to fully complete the Turkish TTSQ because they failed to select answer options and/or unintentionally skipped questions because of problems such as tapping on the text underneath a photograph instead of on the photograph itself and by double-tapping on the next button (see problems 1, 3, 4, and 5 in Table 4). None of the participants noticed they had failed to select answer options or skipped questions while they were completing the questionnaire.

**Table 4:** Frequency and severity of problems encountered during the completion processes for all participants.

Problem	Number of participants who encountered the problem	Number of times the problem occurred	Severity rating
1. Accidentally skipping a screen by double-tapping the “next” button	2	2	Low
2. Double-tapping an answering option causing activation and deactivation of the answer of choice	0	0	—^a^
3. Skipping a screen by accidentally touching the next button with the palm of the hand	0	0	—
4. Not using the navigation function of the photo gallery in question 4 causing the participant to not see all response items	5	22	Serious
5. Touching the text under a photo in question 4 to select an activity, instead of touching the photo itself, causing the activity not to be selected	4	10	Critical
6. Not able to see whether or not a selected answer is activated (not accentuated enough)	1	1	Low
7. Not knowing how to get to the next screen	0	0	—
8. Pushing too hard or tapping too softly on the touch screen so that it does not respond	8	19	Serious
9. Not able to correct a wrong answer	3	3	Medium
10. Not reading the text above the photos in question 5, causing the participant to continue the task given in question 4	1	1	Low
11. Not noticing that the multiple numeric rating scale “effort” scores in question 8 are related to different activities, which in error results in identical scores for different activities	1	1	Low
12. Mistakenly scoring the mirror image in the body chart in question 2	1	1	Low
13. Scoring (serial) questions that do not apply to the participants’ situation (forced by the software)	0	0	—
14. Using navigation function question 4 to try to get to the next screen.	2	2	Low
15. Not knowing how to enter an answer into the TTSQ^b^	2	2	Medium
16. Not being aware of the existence of the “help” function	2	2	Medium
17. Entering more than one answer into an NRS^c^ causing the TTSQ to select only the last entered answer	2	2	Low
18. Activating the “stop” function accidentally by touching it with the palm of the hand holding the tablet	1	2	Medium

^a^This problem was found in the study on the Duth Talking Touch Screen Questionnaire [20], not in this study.

^b^TTSQ: Talking Touch Screen Questionnaire.

^c^NRS: Nummeric Rating Scale

### Stopped Completing Prematurely

Inexperienced Ceyda (age 65 years) read the first question “Do you have pain” (see Multimedia Appendix 1, screenshot 2 “Pain”). She was very doubtful about what answer would be right because her pain had decreased since her first physical therapy visit. She gave back the Turkish TTSQ to the researcher without answering the question because she was not able to decide on her answer, and skipping the question was not a possibility. Afterwards, during interview step 3, she told the researcher that she did not know that the red square with “yes” in it and the green square with “no” in it were “buttons,” which she could have tapped to insert an answer.

Inexperienced Meryem (age 74 years) did not know what to do with the tablet. She read the first question and then spoke directly to researcher MW to give the answer. When the researcher asked her what she thought she should do next, she answered:

Well, I hope to benefit from the therapy. That’s what I am going for

Meryem, age 74 years

When the researcher then asked her if she had any idea what she should do with “the screen,” she seemed to get somewhat nervous and almost whispered:

I don’t know, I do not know what to say

Meryem, age 74 years

Inexperienced Memhet (age 59 years) managed to get to question 4 without encountering any serious or critical usability problems. In this question, he was asked to select photographs of activities in which he was limited (see Multimedia Appendix 1, screenshot 7 “Activity ‘lying”). Memhet tapped on the text beneath the photographs most of the time instead of on the given photographs. He did not notice that this was not sufficient to select the answering option and therefore, thought that he had selected far more photos than he actually had. In question 5, he was asked to select the 3 activities that were most important to him out of those he selected in answer to question 4 (see Multimedia Appendix 1, screenshot 9 “Most important activities”). As most of his answers had not actually been “selected,” he only saw a fraction of his “activity selection.” This confused him. He thought he had misunderstood the question. He did not know how to answer it. After he unsuccessfully tried to skip the question by tapping on the “next” button, he stopped completing the questionnaire by handing it back to the researchers.

### Frequency and Severity of Problems Encountered

Even though 2 participants were able to complete the Turkish TTSQ fully (see Table 3), no participant completed it without encountering any problems. A complete overview of the frequency and severity of all problems encountered during operation of the Turkish TTSQ can be found in Table 4.

### Efficiency

Because of the need to translate the “spoken out loud thoughts” of participants into Dutch, the completion time was lengthened. As a result, the collected data on efficiency were not valid and will not be reported in this paper.

### Satisfaction

#### Positive Remarks

No participant was distinctly positive or negative about the overall ease of use of the Turkish TTSQ. Out of 10 participants, 5 made positive remarks on the way the user interface was designed and on the short completion time.

These visual images are appealing and make it “come to life”.

Imran, age 52 years

Experienced Onur (age 35 years) was positive about the regular overviews of given answers, and inexperienced Eren (age 48 years) was positive about the short length of the questionnaire.

#### Recommendations for Improvement

Out of 10 participants, 9 formulated recommendations for improvement. Most mentioned recommendations were improved accentuation of the activated response items, give a complete overview of activities to choose from in answer to question 4, and shorten the instruction clips by limiting the information to the main issues.

Inexperienced Seyda (age 65 years) and Meryem (age 74 years) had trouble concentrating on the information in the introduction clip, as did others. However, they were not sure if limiting the amount of information or length of the clip was going to help them. They felt it would just be too difficult for them to learn to work with the Turkish TTSQ, regardless of improvements on its usability. They linked their lack of ability to comprehend and remember the instructions given on their lack of experience with operating tablet computers, their older age, and their health status.

Experienced Berat (age 38 years) recommended limiting the text above the overviews. For example, he suggested deleting the first sentence from the text: “On this screen you see all the activities that you selected in previous screens. These are the activities in which you are limited. Is that right?” (see Multimedia Appendix 1, screenshot 8 “Overview activities”).

Some participants suggested adding more advanced options to the Turkish TTSQ. Experienced Onur (age 35 years) and Berat (age 38 years) recommended a swipe function for the screens that contained rows of activity photos. Experienced Elif (age 40 years) would have liked to see muscles in the body chart so she would be able to indicate the location of her pain more precisely. Like Elif, Onur and inexperienced Eren (age 48 years) also wanted to be able to indicate the locations of their complaints more precisely, but they suggested a function that would enable them to zoom in on a specific body part.

## Discussion

### Principal Findings

In all, 2 participants, who had prior experience with using tablet computers, managed to complete the questionnaire fully without leaving any parts unanswered. No participant in this study was able to complete the questionnaire without encountering a usability problem.

A total of 17 different kinds of problems were found. Three problems should be addressed during future development of the tool based on their severity score [40]: “Not using the navigation function of the photo gallery in Question 4 causing the participant to not see all presented response items,” “Touching the text underneath a photo in Question 4 to select an activity instead of touching the photo itself causing the activity not to be selected,” and “Pushing too hard or tapping too softly on the touch screen causing the touch screen to not respond.”

No participant was distinctly satisfied or dissatisfied about the overall ease of use of the Turkish TTSQ. Positive remarks were mainly made on the user interface and the short completion time of the Turkish TTSQ. The most frequently made recommendations were improve accentuation of the activated response items, give a complete overview of activities to choose from in answer to question 4, and shorten the instruction clips by limiting the information to the main issues. Two inexperienced participants felt that, regardless of what improvements might be made, it would just be too difficult for them to learn to work with the device.

### Strengths and Limitations

A strength of this study was the inclusion of 10 members of a target population that is generally “hard to reach” for researchers, including some of the most vulnerable subjects within this population [42]. This made it possible to both collect data from people who are rarely represented in research populations and, at the same time, gather knowledge about the effects strategic and methodological choices have on the quality of research in such populations. Researcher SH played an important role in the recruitment and data collection within this study. His Turkish background and his being a native Turkish speaker, combined with his network, status, and trustworthiness as a physical therapist working in the community, may have had a positive influence on the willingness of potential candidates to participate in this study [43]. This hypothesis is reinforced by the fact that, although recruitment was done in 12 different physical therapy practices, 8 out of 10 participants were recruited in the practice where researcher SH was employed.

The positive effect researcher SH had on the sampling procedure may also have had a downside. Despite all efforts of the researchers to inform potential participants thoroughly and make sure that participation was done voluntarily, the authority of researcher SH as researcher and physical therapist [43] may have caused participants to agree to participate too quickly without really foreseeing what was being asked of them. The majority of the participants seemed to have “a lot on their plate” and were, therefore, not able to entirely focus on their tasks during the data collection process. A total of 8 out of 10 participants reported multiple health problems. One participant even ended the interview prematurely because it became too much for her because of her physical and mental state. Another participant, who reported 11 different kinds of health problems, told the researchers that his biggest problem was not even his health status but his poor financial situation. In hindsight, the researchers got the impression that, for some, participation in this study may have been too much to ask.

The bilingual research setting also brought some limitations to this study. Apart from the translation lengthening the completion time, 3 participants forgot to insert some of their answers during the completion process, although they *did* formulate their answers when thinking out loud. They all said they would not have forgotten this in a “real life” physical therapy setting where there would have been no observers or interpreters present and they would not have been asked to think out loud. A total of 3 other participants said that the translation limited their ability to concentrate on their task and thoughts. This may have caused participants to make more mistakes than they would have done had the whole interview been in the Turkish language.

### Comparison With Prior Work

Although there is a considerable amount of overlap in the kind and severity of problems encountered in the current and Dutch TTSQ study [20], the participants of this study encountered different kinds of problems and were less able to complete the questionnaire fully than those in the Dutch TTSQ study. The explanation for this can be found in the fact that, compared with the Dutch study, the population of this study was less educated, had lower health literacy, and had less experience with using tablet computers. In this study, no participant was completely satisfied or dissatisfied with the overall ease of use of the Turkish TTSQ, although, in the Dutch TTSQ study, the participants were not only very satisfied but their expectations of ease of use of the tool were exceeded [20]. In contrast to the Dutch TTSQ study, not all Turkish participants had the sense of self-efficacy to be able to complete the Turkish TTSQ, no matter what improvements might be made. The results of the Dutch TTSQ study showed that participants with lower education and less experience in using mobile technology were less able to operate it effectively [20]. This is confirmed by the results of this study.

Two earlier studies were found in which usability was part of the assessment of a direct translation of a Talking Touchscreen (TT) questionnaire, both published by Hahn et al [44,45]. In the 2003 study, the usability components “satisfaction” and “efficiency” were tested. In this study, 30 Spanish-speaking patients with cancer completed a TT which contained the Functional Assessment of Cancer Therapy-General (FACT-G) [46] and the Short Form-36 Health Survey [47]. A total of 50% (7/15) of the participants had lower than 7th grade education. Satisfaction with ease of use and efficiency were tested by presenting evaluation questions on the use of the TT followed by a short debriefing interview. What is noticeable about the satisfaction and efficiency results is that all 30 participants reported that they thought of the tool as “very easy” or “easy to use” and the completion “did not take too long,” whereas 57% (8/15) of participants with less than 7th grade education and 14% (2/15) of the participants with more than 7th grade education preferred an interviewer orally conducting the questionnaire to use of the TT. Hahn et al [44,45] interpreted these results in a positive way and reported that *many patients either preferred using the touchscreen rather than having an interviewer ask the questions, or had no preference.* Although true for the more educated participants, the majority of the less educated participants did not prefer using the TT. Hahn et al [44,45] concluded their paper by stating that *the “Talking Touchscreen” will allow Latino patients with varying literacy skills to be included more readily in clinical trials, clinical practice research and QOL studies.* This conclusion may be too 1 dimensional, given the results they reported and the methods they used. In the other study by Hahn et al, published in 2010, only user satisfaction was tested [45]. In this study, 414 Spanish-speaking patients with cancer were included of which 213 had low levels of literacy. The tested touch screen system contained the FACT-G [46], SF-36 [47], and Standard Gamble Utility Questionnaire [48]. The methods used to test satisfaction about the ease of use were highly comparable with the earlier study of Hahn et al [44]. Looking at the quantitative results, one can conclude that, although satisfaction among the majority of the participants was high, low-literacy participants were less satisfied with the ease of use of the TT than were those with high literacy. It is hard to compare the results of the studies of Hahn et al with the results of this study because, although the participants in their studies could ask for assistance from the researchers during completion of the TT, participants in this study did not receive any help at all. In the 2003 study, 60% of participants received help from a researcher during completion of TT; how many received help in the 2010 study was not reported. It can be concluded that researchers in this study tested and reported the usability of their tool much more thoroughly. Although it is difficult to directly compare the results of the Hahn et al studies with the current studies because of differences in study setups and the detail in which results were reported, the results of both Hahn et al studies seem to confirm our findings that it is harder for less educated participants to use a TT than for higher educated participants.

### Conclusions

Just like the Dutch TTSQ, the Turkish TTSQ needs improvement before it can be released. The results of this study confirm the conclusion of the Dutch TTSQ study that participants with low education and little experience in using mobile technology are less able to operate the TTSQ effectively. Although the methodology of this usability study was very thorough, using a Dutch-speaking interviewer and Turkish interpreter has had a negative effect on data collection.

### Directions for Future Research

The aim of the project, of which this study is a part, is to create multiple language versions of the TTSQ to help Dutch physical therapy patients, regardless of their level of health literacy, to elucidate their health problems and limitations, and set treatment goals. The results of both usability studies of the TTSQ show that this should particularly be improved for the least skilled future users. Therefore, the logical next step is adapting and testing both language versions of the tool solely with inexperienced users who have low literacy. When the pretests show that future users at risk of exclusion are able to complete the Turkish and Dutch versions of the TTSQ fully without encountering serious or critical usability problems, pretests on response processes should be conducted to get a first impression of the face validity of both versions of the questionnaire [49]. In addition, the equivalence of both language versions should be tested using item response theory [50]. Dependent on the results of these response processes and item response theory studies, cultural adaptation of the Turkish TTSQ may be needed to avoid bias from cultural and linguistic effects on interpretation, retrieval, judgment, and response selection, which are the 4 phases of the response process as described by Tourangeau et al [51]. Both researchers and participants should communicate in Turkish in all future studies on the Turkish TTSQ to avoid the methodological problems encountered in this study. Recruitment of participants with a Turkish background should be done by intermediaries with Turkish backgrounds, rather than by the researchers themselves, to limit the chance of people agreeing to participate too easily without foreseeing the consequences of their participation. When the results of all pretests are satisfactory, the last step in research should be quantitative usability, validity, and reliability testing to produce generalizable data.

No data on levels of literacy, health literacy, or digital skills are available for the Turkish minority group in the Netherlands. Research should be done to get insight into these characteristics and into attitudes toward use of information and communication technology in general and of mobile health (mHealth) technology more specifically within this and other minority groups. Otherwise, these already disadvantaged groups may not be able to profit from the advantages of the use of mHealth and electronic health technologies [52-54]. This may add to the ongoing exacerbation of health inequalities in the Netherlands [55].

It is of great importance to keep striving for the development of TT questionnaires, which are user-friendly to low literacy minority patients who have not mastered the native language of the countries in which they are living in. Such tools will greatly facilitate data collection within these hard-to-reach populations. It will empower vulnerable patients who will be able to give their input to research and clinical practice. And because they will not need help or instructions from researchers or health care providers, it will reduce staff burden, costs, and interviewer bias. The use of TT questionnaires may also serve as a way to increase exposure of underserved populations to new technologies and contribute to information about the experiences of diverse populations with these technologies [56]. To get reliable and valid test results for the evaluations of these tools, researchers need to keep striving for research setups and methods that fit the needs and abilities of hard-to-reach populations. Publishing positive as well as negative results on usability, reliability, and validity and giving as much insight into evaluation methods, study contexts, and setups as possible will help researchers and developers in finding ways to accommodate hard-to-reach populations and contribute to the body of knowledge on inclusive design-oriented research.

## Appendix

Multimedia Appendix 1 Screenshots Turkish Talking Touch Screen Questionnaire.

## Abbreviations

FACT-G: Functional Assessment of Cancer Therapy-General
mHealth: mobile health
SBSQ-D: Set of Brief Screening Questions-Dutch version
TSTI: Three-Step Test-Interview
TT: Talking Touchscreen
TTSQ: Talking Touch Screen Questionnaire
