# Supplementary material for: Usability of the Turkish Translation of the Dutch Talking Touch Screen Questionnaire for Physical Therapy Patients With a Turkish Background: Qualitative Study
Source: JMIR Form Res. 2020 Feb 13;4(2):e14189. doi: 10.2196/14189 (PMC7055804; doi:10.2196/14189)

Multimedia Appendix 1

Screenshot 1 'Welcome'
Introduction movie

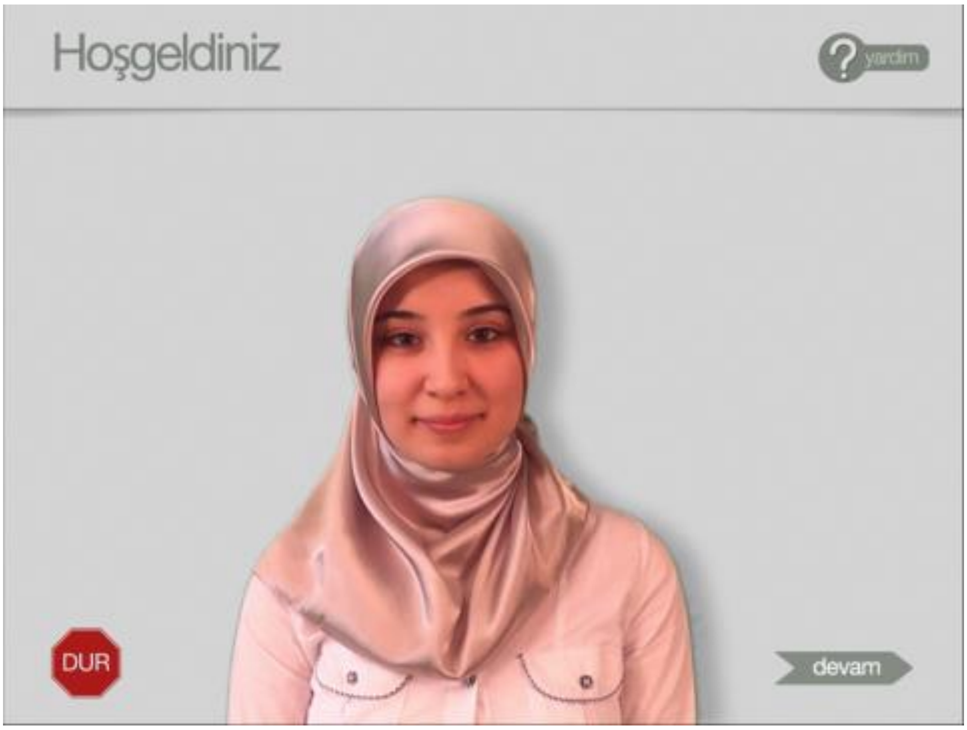

Screenshot 2 'Pain'
Question 1: "Do you have pain? Yes/No"

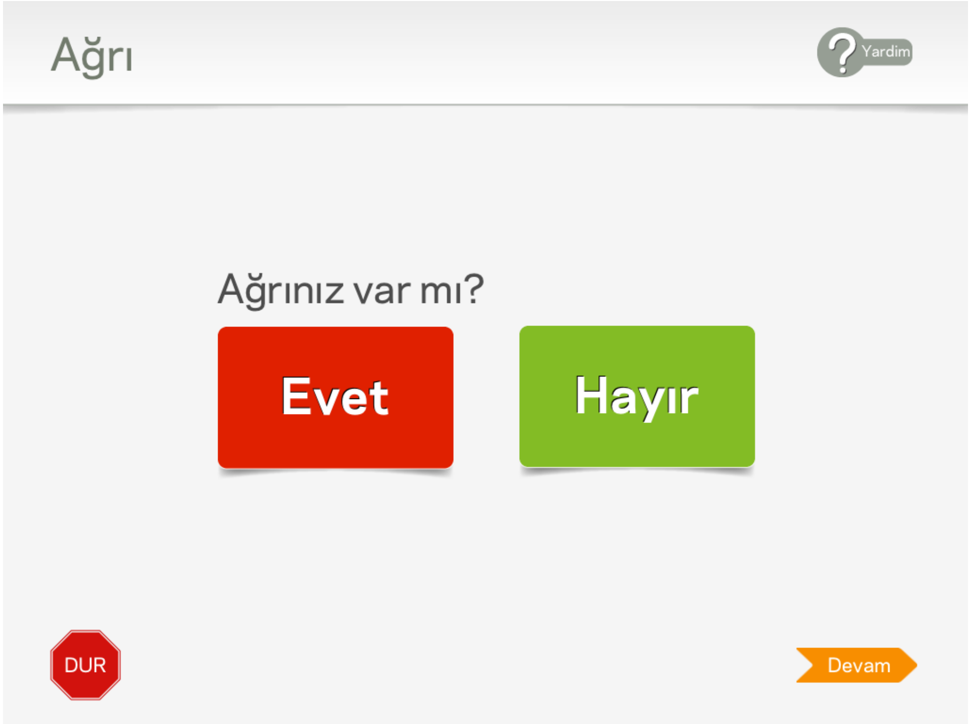

Screenshot 3 ‘Location of the health problem’

Question 2: “Tap on the location of your health problem. You can tap on multiple locations.”

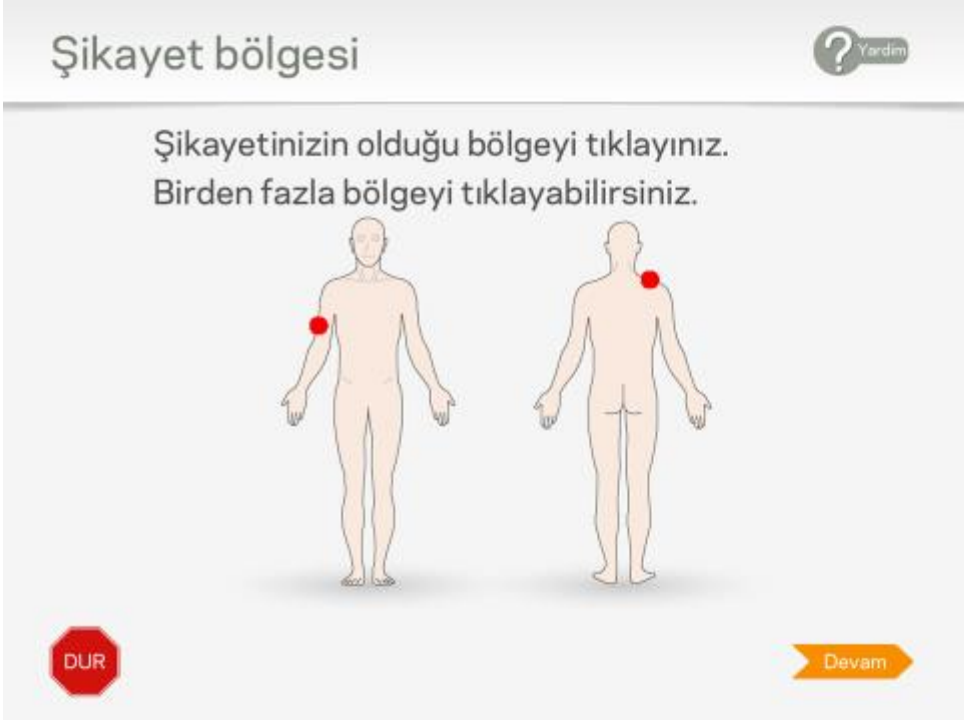

Screenshot 4 ‘pain severity’

Question 3: “This is the location of your pain.
Rate the severity of your pain on the scale below.”

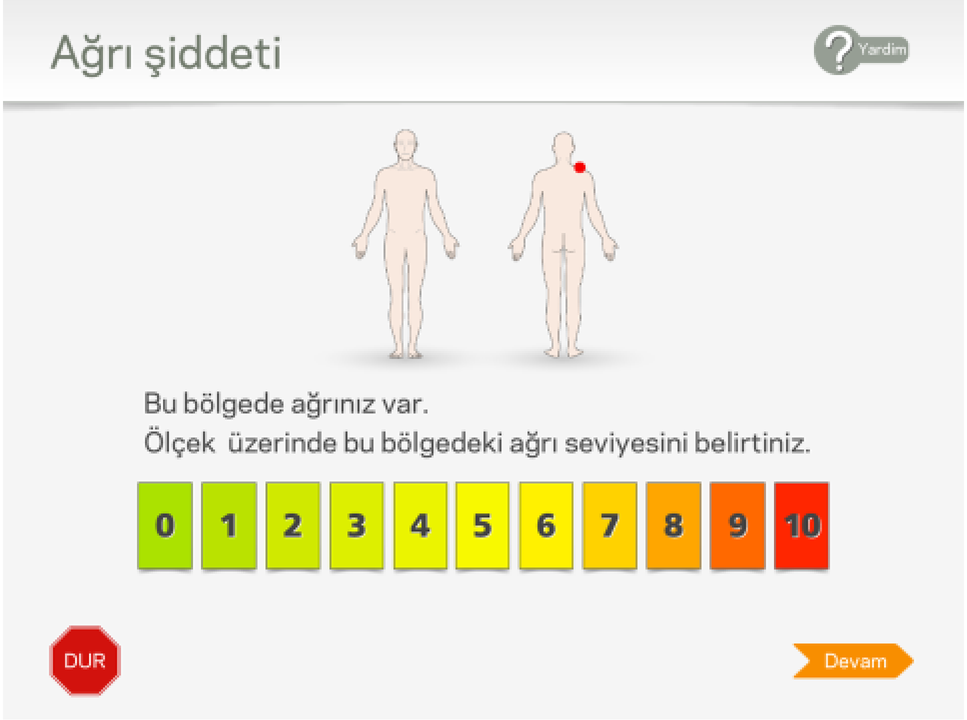

Screenshot 5 ‘Overview location of the health problems’
Overview answers question 1-3:
“This is the location of your health problems.”

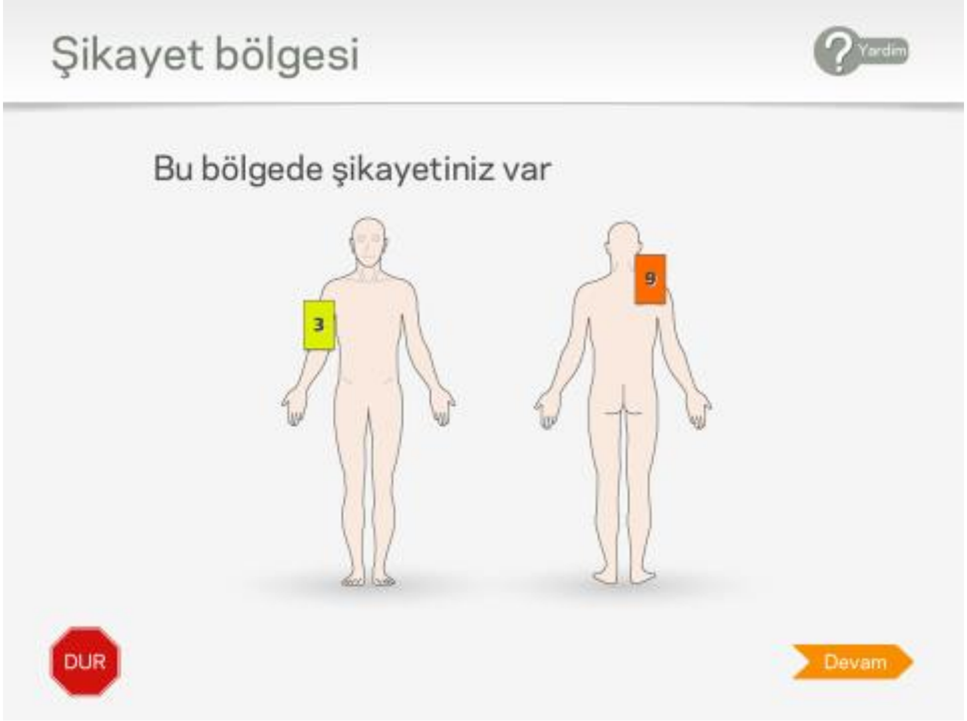

Screenshot 6 ‘Activities’
Instruction movie question 4

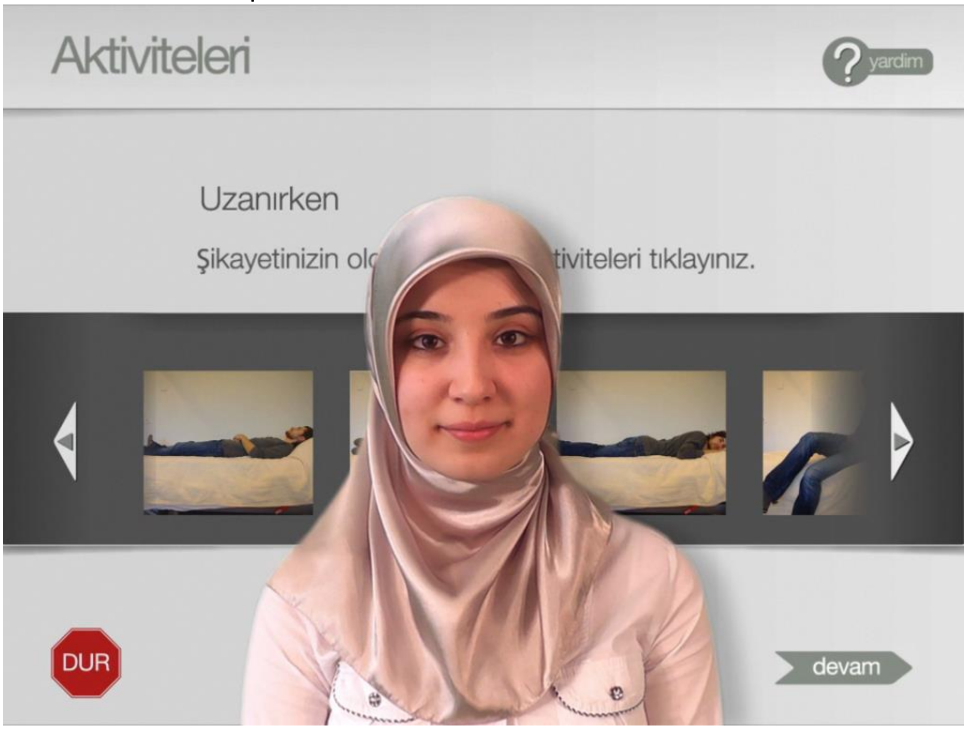

Screenshot 7 Activity 'lying'

Question 4: "Select the activities in which you are impaired"

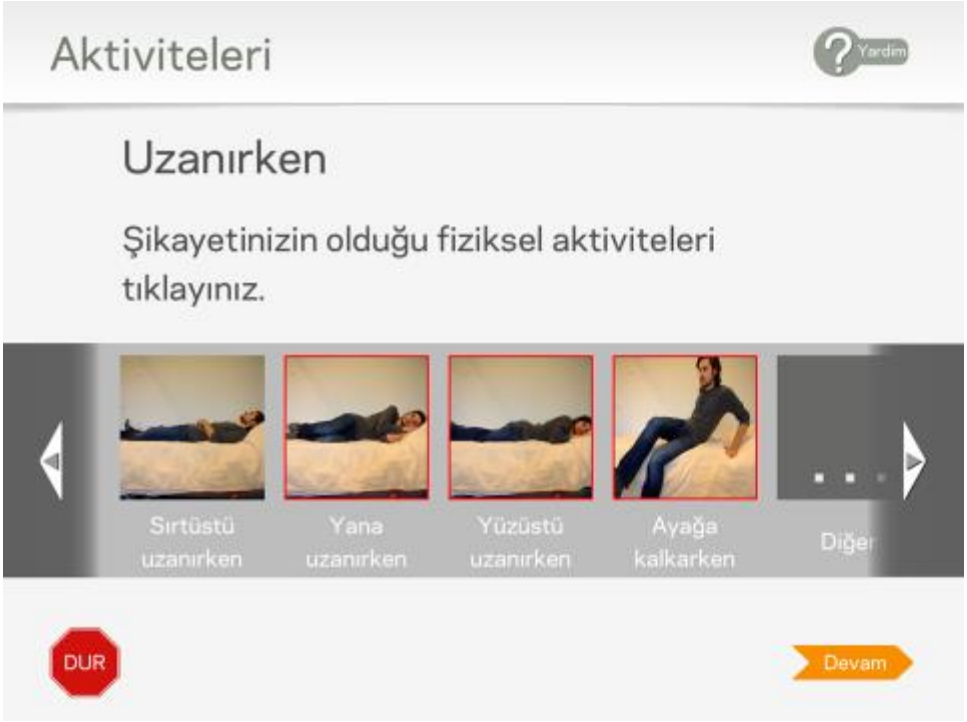

Screenshot 8 'Overview activities'

Overview answers question 4:

"On this screen you see all the activities that you selected in previous screens. These are the activities in which you are impaired."

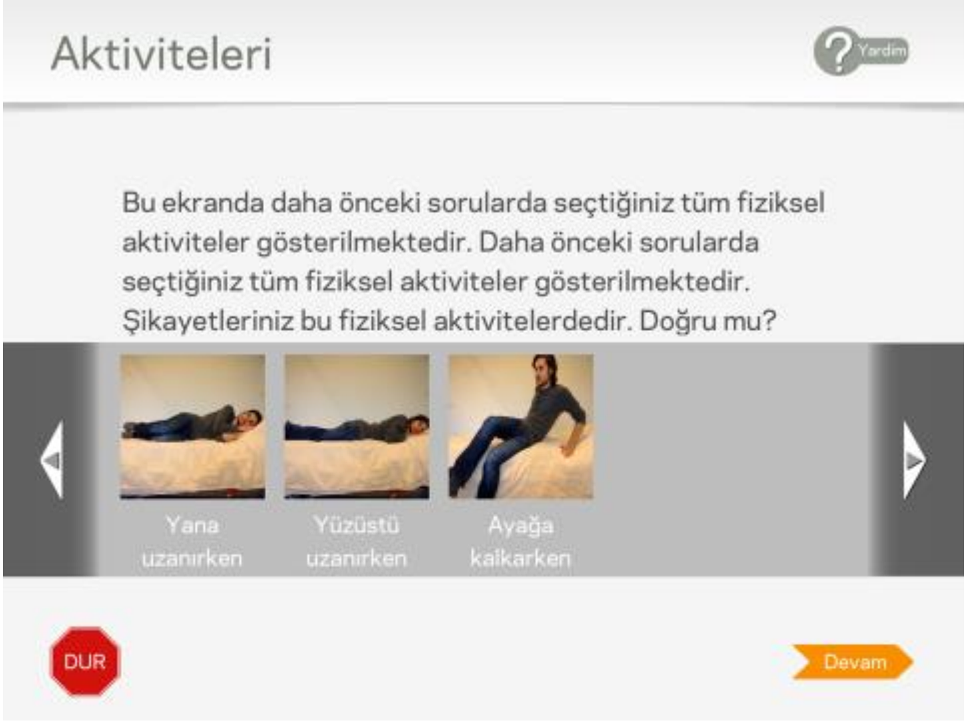

Screenshot 9 ‘Most important activities’
Question 5: “Select the three activities which are most important to you”

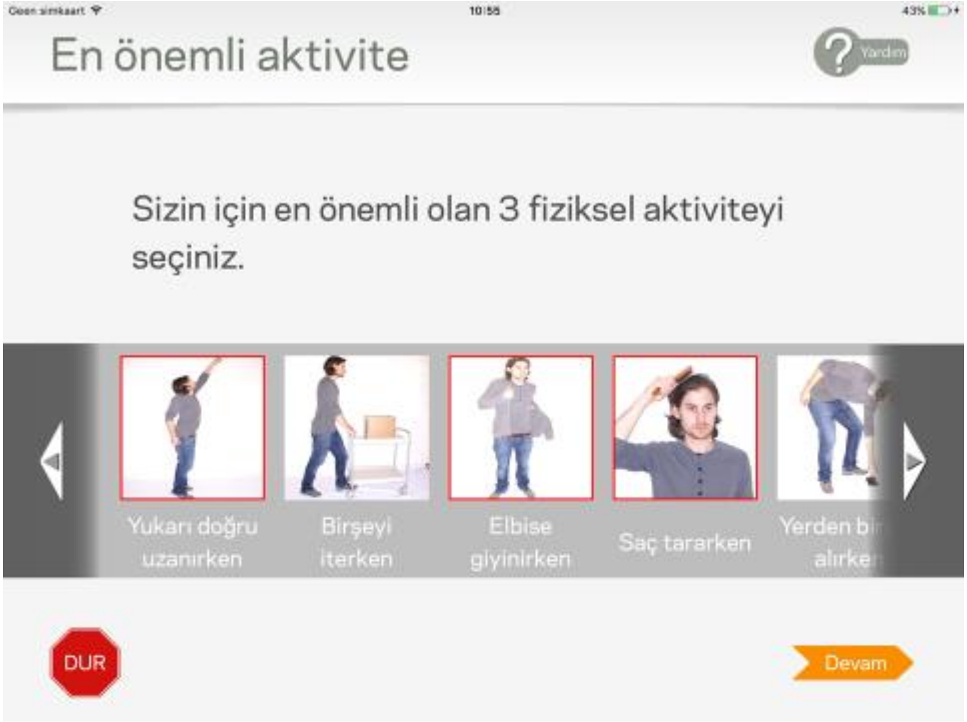

Screenshot 10 ‘overview most important activities’
Overview answers question 5:
“You chose these three activities. Is this correct?”

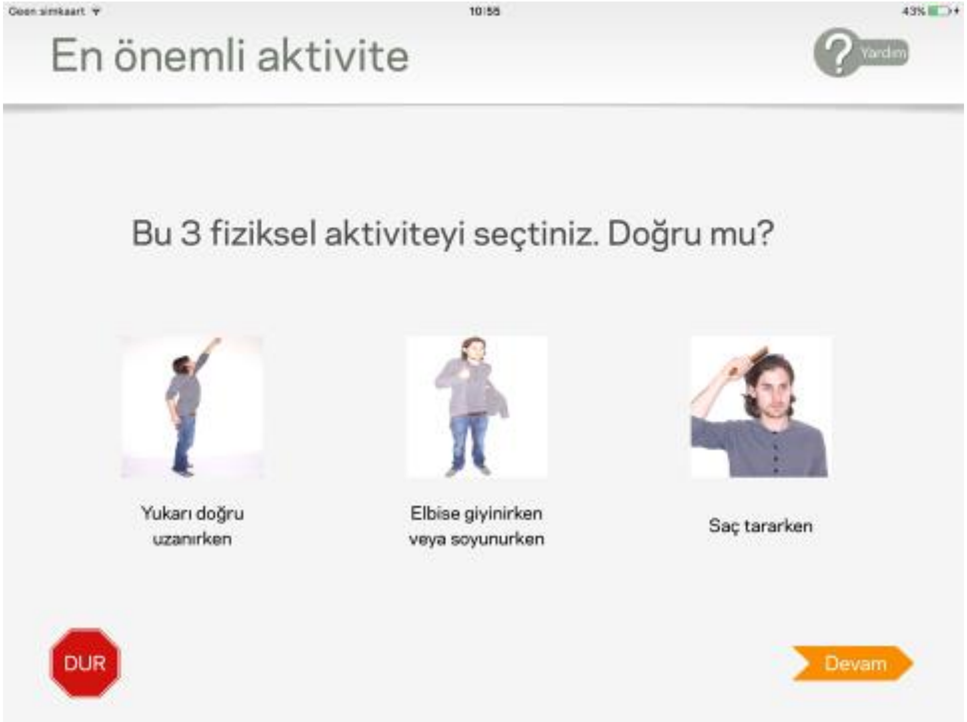

Screenshot 11 'Most important activity 1'

Question 6: "Select the activity which is most important to you"

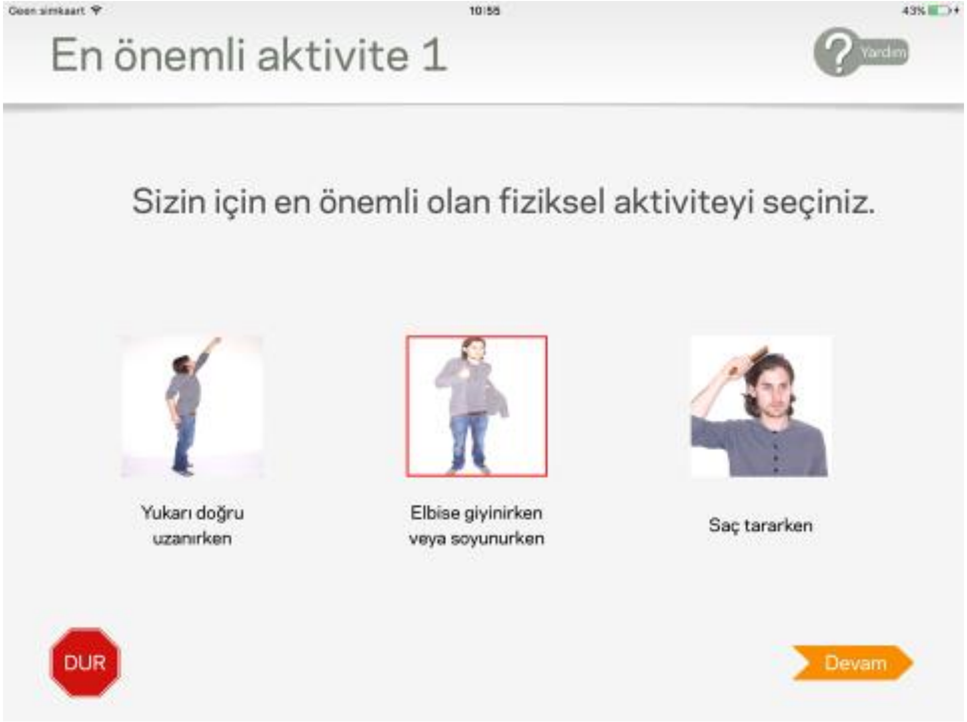

Screenshot 12 'Most important activity 2'

Question 7: "Which of these two activities is still most important for you now?"

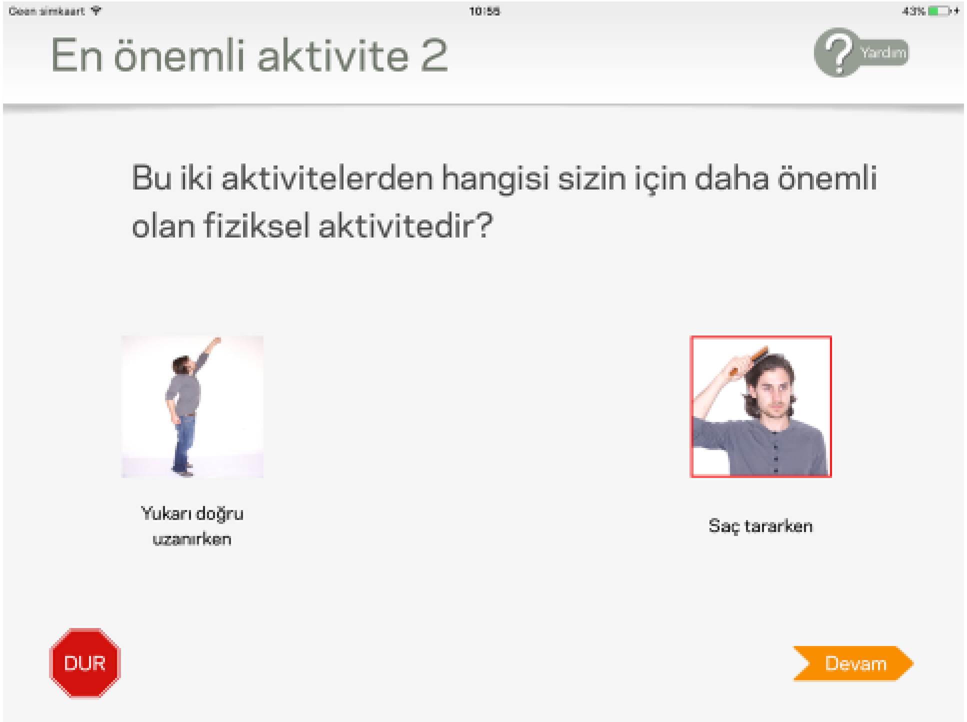

Screenshot 13 ‘Effort activity 1’
Question 8: “Rate the effort it takes to carry out this activity“

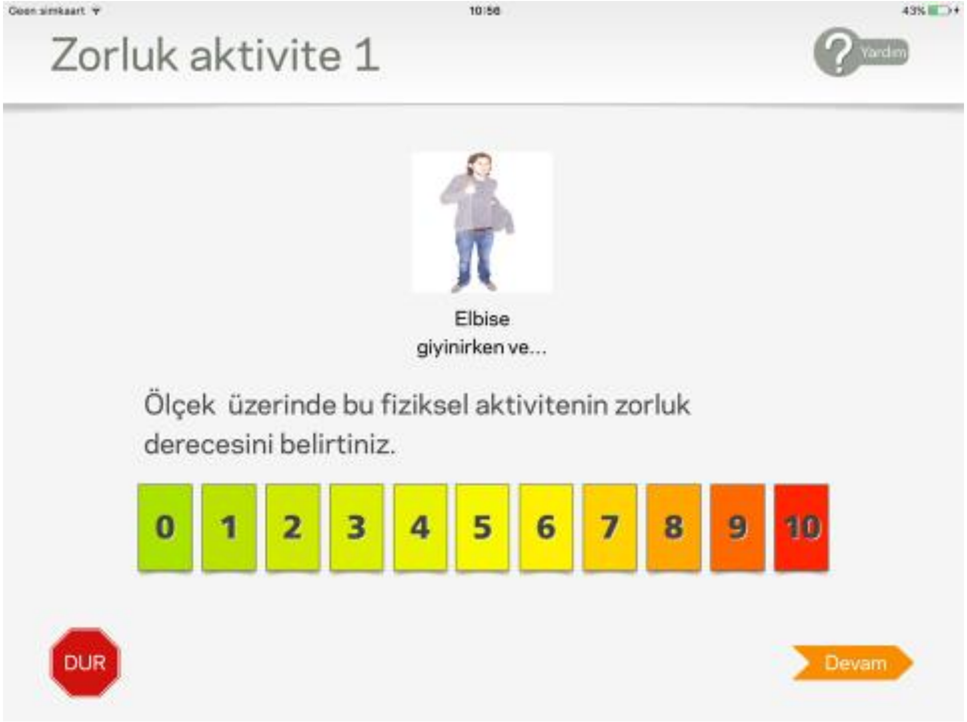

Screenshot 14 ‘overview most important activities and effort’
Overview answers question 6-8:
“On this screen you see the activities that are most important to you in order of most important to least important. Is this correct?”

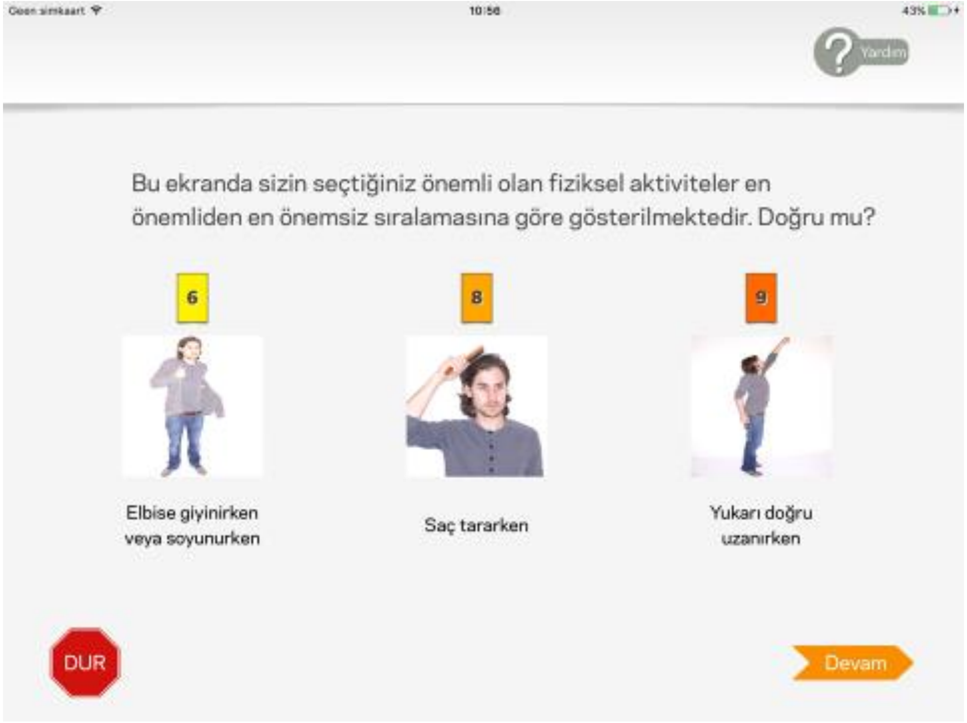

Screenshot 15 ‘overview all outcomes of the questionnaire’
Overview answers total questionnaire:
“On the screen you see an overview of all your answers you provided until now.”

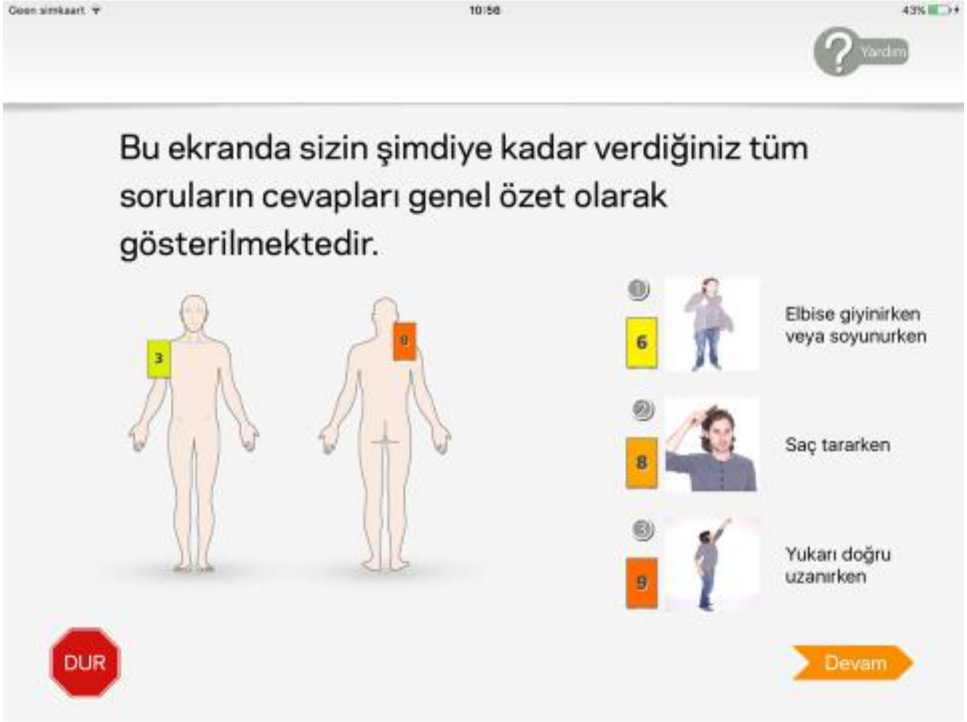

Screenshot 16 ‘Thank you’
Closing movie

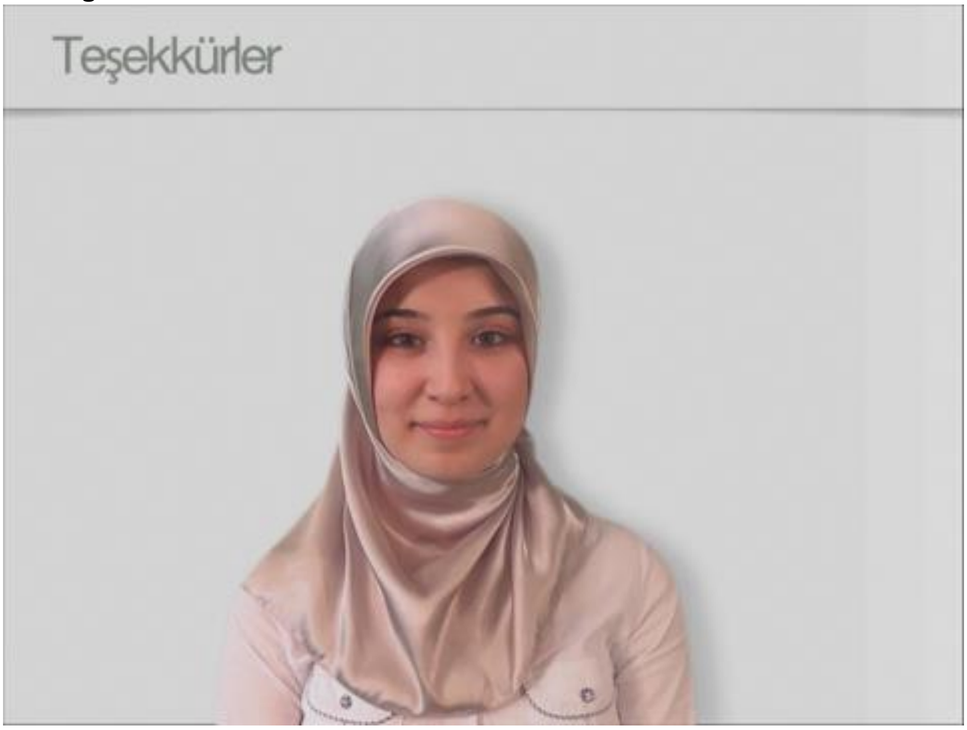

Supplement: Multimedia Appendix 1 [file formative_v4i2e14189_app1.pdf]
